# Malaria trends in Silt’i district from 2009-2015 and current childhood malaria in K’ibbet hospital, south-central Ethiopia

**Published:** 2017-12-01

**Authors:** Sani Dedgeba, Hassen Mamo

**Affiliations:** 1 K’ibbet Secondary and Preparatory School, K’ibbet, Ethiopia; 2 Department of Microbial, Cellular and Molecular Biology, College of Natural Sciences, Addis Ababa University, Addis Ababa, Ethiopia

## Abstract

**Background:**

Regular evaluation of the magnitude of malaria in children in a given locality is important to devise targeted control interventions. This study was conducted to assess current malaria infection among children (0-14 yrs) and trends in malaria between 2009 and 2015 in Silt’i district in south-central Ethiopia.

**Materials and methods:**

Febrile children (body temperature ≥37.5°C) visiting the K’ibbet hospital between September 2015 and January 2016 were enrolled. Finger-prick blood samples were collected, smears prepared, Giemsa-stained and examined. In addition, past (2009-2015) retrospective malaria data was reviewed. Descriptive statistics was used to analyse the data.

**Results:**

In the current cross-sectional survey, 43 (4.3%) children (n=1007) were diagnosed with malaria. Of these, 35 (81.4%) were *Plasmodium vivax* and only 8 (18.6%) *P. falciparum*. All cases were mono-infections. Concerning health data on record, overall 47,467 malaria-slide-confirmed cases were recorded (45.8% *P. vivax* and 54.2% *P. falciparum*), with a substantial decline in malaria between the baseline in 2009 (15,141 cases) and 2015 (821 cases).

**Conclusions:**

The findings demonstrated that malaria in children was continuously declining in the study area. However, control interventions must be maintained and scaled-up to sustainably protect children as well as the general population and eventually eliminate the disease from the locality and country as a whole.

## 1 Introduction

Children, particularly those younger than five years of age, including new-borns and infants less than 12 months of age comprise the most vulnerable groups affected by malaria. They bear about 69% of overall malaria-related deaths [1]. In high transmission areas infants become vulnerable to malaria at approximately 3 months of age, when immunity acquired from the mother starts to wane and are at increased risk of rapid disease progression, severe malaria and death thereafter. Severe anaemia, hypoglycaemia and cerebral malaria are features of severe malaria that are particularly common in this age group. *Plasmodium vivax* malaria, which is traditionally viewed as relatively benign, is emerging as a major cause of morbidity and mortality in infants and young children [2-4].

About 75% of the total landmass of Ethiopia is malarious and 60% of the population is at risk of the disease [5]. Ethiopia fact sheet of health statistics 2016 based on WHO 2015 [1] delineates the following regarding malaria: Under-five death by malaria in 2000, 2005 and 2010 was 2.0%. The number of these children with fever who received treatment with any anti-malarial drug in 2014 was 26.0%. In that same year the proportion of these children sleeping under bednets was 30.0%.

Although there have been major improvements in the malaria situation challenges remain ahead. The Federal Ministry of Health (FMoH) annual performance report [6] indicated that between September 2014 and August 2015 a total of 2,174,707 malaria cases (confirmed plus clinical) and 662 deaths were documented with case fatality rate of 0.03%. The highest number of total malaria cases was reported from Amhara (610,486) followed by Oromia (430,969) and Southern Nations, Nationalities and Peoples Region (SNNPR) (375,746). Although Ethiopia is in the malaria control phase it has set a plan to eliminate malaria in selected low-transmission settings by 2020 [7]. The SNNPR includes some of the southern and southwestern parts of Ethiopia. The region has an area of 110,931 km^2^, which is approximately 10% of the country’s total area [8]. About 26% of the population in the region is at-risk of malaria and the disease is a primary cause of outpatient and inpatient consultations and hospital deaths [9]. According to the FMoH annual report 2015 [6], in SNNPR between September 2014 and August 2015 the population at-risk of malaria was 12,238,426. In Silt'e Zone which is one of the administrative Zones in SNNPR, a total of 221,458 bednets were distributed out of 391,575 required.

Malaria remains one of the major health problems in Silt'e Zone. Out of eight districts and one town administration in the zone five (Dalocha, Silt'i, Lanfuro, Sankura and Wulbareg) are malarious. In 2009, 8.5% of Silt'i district population was malaria slide-positive (district health office record) showing the high public health burden of the disease in the population. However, few published reports are available on recent malaria impact among children, and its seasonal or annual trend in Silt'i district. Both FMoH and SNNPR reports provide little information about detailed malaria status of districts within zones. Evaluation of the levels and time trends in malaria burden are crucial for assessment of progress towards goals and planning national health services and focusing future efforts. The present study therefore aimed at estimating the prevalence of malaria in Silt'i district over the period 2009-2015 and also the current situation (beginning of 2016) of the malaria burden in children (0-14 yrs).

## 2 Materials and methods

### 2.1 Study area

The study was conducted in Silt’i district in SNNPR. The main rainy season in the district extends from June to September with annual rainfall of 650-1800mm. The district is bordered on the south by Lanfuro and Dalocha, and in the southwest by Alicho werero, which are sister districts in the zone, on the north by Gurage zone and on the east by Oromia Region.

Silt’e zone is generally considered malarious with all the 704,835 of its population at-risk [9]. The aforementioned same regional health bureau document [9] shows that LLIN coverage was 94.0% (1 LLIN per 1.8 people) for the zone, which was indeed among the lowest compared to the coverage in other zones or special districts in the region. The Silt’i district has a total population of 177,249 [10], and is the leading malaria hotspot in the zone.

Specifically, the study was based in K’ibbet town, 147 km to the south of Addis Ababa, which is the administrative centre of Silt’i district. The climatic condition of the town is considered a midland with mean monthly temperature of 20-25^0^C. K'ibbet town has a total population of 7134 (3667 males, 3467 females) in two kebeles (the lowest administrative unit perhaps divided into villages). At the time of this study K’ibbet hospital was the only hospital in the district, and there were five clinics in the town. The hospital was established in 2014 having a catchment area of 13 kebeles. During this study period the hospital was serving 62,251 people from K’ibbet 01 and 02 kebeles and neighbouring kebeles Senena Gerara, Weleya Sidist, Koto and Lobrera.

The Silt’i district health office report shows that acute respiratory infections, malaria, leishmaniasis, diarrhoea and intestinal parasites are major health challenges in K’ibbet town. The health facility record indicates that malaria is among the leading causes of morbidity in the town’s surrounding rural villages, which have more favourable climate for mosquito vectors although the town *per se* is a midland. The common malaria control activities - prompt diagnosis and treatment, bednet use, indoor residual spraying (using propoxur or bendiocarb), and environmental management (mosquito larval source management) were well underway in the town and its neighbourhood. LLIN coverage was 95% (for households having 2, 3-5 and 6-9 members, with 1, 2 and 3 LLINs, respectively).

### 2.2 Blood sample collection and analysis

Blood slides were taken from all febrile children at K’ibbet Hospital emergency or outpatient departments, and examined by specialist microscopists in the hospital following an established protocol [11]. Briefly, finger-prick blood samples were collected, and thick and thin smears prepared. The thin smear was fixed with methanol, flooded with Giemsa stain and left for 10 minutes. The slide was washed thoroughly under tap water, left to dry and backside cleaned with cotton wool. The thick film was flooded with Giemsa stain and allowed to stand for 30 minutes. The slide was washed backside wiped with cotton wool and placed in a draining rack to air dry. Both types of slides were examined, malaria parasites detected and species identified.

### 2.3 Data analysis

Data were checked for completeness and consistency, and entered into statistical package for social sciences (SPSS) version 20 (IBM SPSS). The Chi-squared (X^2^) test was used to test differences in malaria prevalence between years, seasons (months), sexes, and age groups for both retrospective and current cross-sectional data.

### 2.4 Ethical approval

Ethical approval was obtained from Silt’i district health office to use the described retrospective malaria morbidity data and the current cross-sectional survey.

## 3 Results

**Figure 1:**
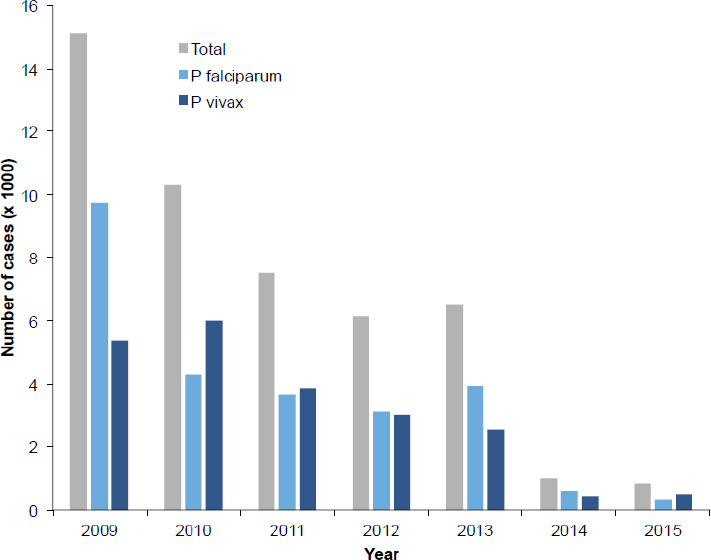
Annual number of malaria positive patients in Silt’i district between 2009 and 2015.

Among patients diagnosed for malaria between January 2009 and December 2015, a total of 47,467 were slide-positive. On average, 6781 slide-confirmed cases visited Silt’i district health facilities each year. There was successive reduction in malaria prevalence from 2009 onwards except in 2013 when the number was little higher than in 2012. However, the number of total examined patients each year, and malaria distribution by age or sex were not available in the district health office making it impossible to calculate malaria prevalence in terms of sex and age groups. Also, seasonal distribution was lacking. On the whole; 25,722 (54.2%) and 21,745 (45.8%) of the cases were attributed to *P. falciparum* and *P. vivax* mono-infections, respectively (Table 1, Figure 1) with no statistically significant difference (p = 0.996). There were more *P. falciparum* mono-infections in 2009 (64.4%) and 2013 (60.7%) than *P. vivax* (35.6% and 39.3%, respectively), and the reverse was observed in 2010 and 2015.

**Table 1:** Slide-confirmed malaria cases (2009-2015) by *Plasmodium* species from Silt’i District Health Office.

Year	Slide-positive* n (%)	*P. falciparum* n (%)	*P. vivax,* n (%)	p-value
2009	15141 (31.9)	9754 (64.4)	5387 (35.6)	
2010	10321 (21.7)	4315 (41.8)	6006 (58.2)	
2011	7514 (15.8)	3653 (48.6)	3861 (51.4)	0.002
2012	6142 (12.9)	3125 (50.9)	3017 (49.1)	
2013	6505 (13.7)	3951 (60.7)	2554 (39.3)	
2014	1023 (2.2)	596 (58.3)	427 (41.7)	
2015	821 (1.7)	328 (40.0)	493 (60.0)	
Total	47467 (100)	25722(54.2)	21745(45.8)	0.996

*The total number of slides examined was not available.

**Figure 2:**
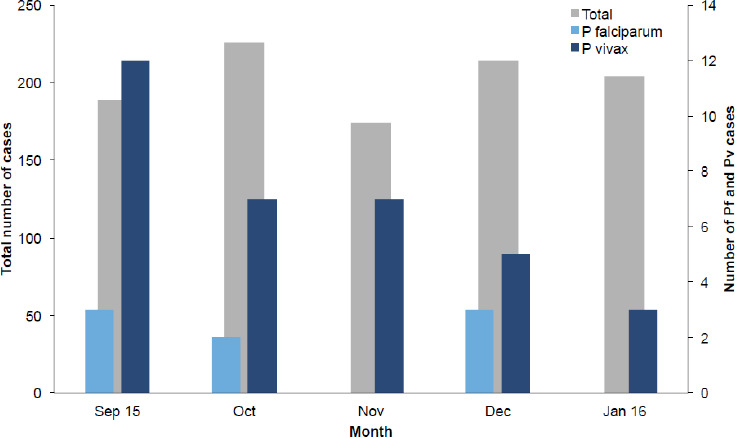
Monthly profile of malaria-positive children (0-14 yrs) at K’ibbet hospital, September 2015-January 2016.

Among 1007 febrile children diagnosed for malaria between September 2015 and January 2016, 43 (4.3%) were slide-positive. On average 201 febrile and 9 malaria-confirmed cases visited K’ibbet Hospital each month. The average monthly malaria prevalence was 4.5%. However, the number of suspected and confirmed cases showed a fluctuating pattern in the months studied (Table 2). Much higher number of cases (31 (72.1%)) were registered in September through November shortly after the rainy season. Only 12 of the cases (27.9%) were observed during the driest months (December-January 2016). The number of confirmed cases in January 2016 was lower than that in September 2015. Similarly, a higher number of febrile children visited the hospital in October than November 2015. In general, there was a decline in the number of confirmed cases of malaria in January 2016 compared to the previous four months indicating the seasonal trend although cases existed throughout the dry season (Figure 2). There were only 6 cases from K’ibbet town. The other 37 cases were from rural kebeles (Senena Gerera 12, Koto 5, Weleya sadist 7, Lobrera 4, others 9). No severe malaria severe cases or malaria-related deaths were encountered.

**Table 2:** Seasonal pattern of malaria in suspected and slide-confirmed children by *Plasmodium* species in K’ibbet hospital.

Month	Total examined	Slide-positive n (%)	*P. vivax* n (%)	*P. falciparum* n (%)	p-value
Sept ‘15	189	15 (7.9)	12 (80.0)	3 (20.0)	
Oct	226	9 (4.0)	7 (77.8)	2 (22.2)	0.059
Nov	174	7 (4.0)	7 (100)	0 (0.0)	
Dec	214	8 (3.7)	5 (62.5)	3 (37.5)	
Jan ‘16	204	4 (2.0)	3 (37.5)	1 (62.5)	
Total	1007	43 (4.3)	35 (81.4)	8 (18.6)	

On the whole, 35 (81.4%) and 8 (18.6%) of the malaria cases were attributed to *P. vivax* and *P. falciparum* mono-infections, respectively. There were no mixed infections during the study period. *P. vivax* was consistently more prevalent than *P. falciparum*, irrespective of season (Table 2). In terms of sex, 571 (56.7%) of the total number of children examined were males and 436 (43.3%) females. Of total slide-positives, 51.2% were males and 48.8% females (Table 3). Malaria slide-positives among males were 22 (3.9%) and among females 21 (4.8%). The data showed that more males were examined than females, but there was little variation in malaria positivity by sex.

**Table 3:** Age and sex distribution of malaria suspected and slide-confirmed cases at K’ibbet Hospital, Silt’e Zone, SNNPR, Ethiopia, September 2015-January 2016 (n=1007).

Sex	Examined n (%)	Slide-positive n (%)	*P. falciparum* n (%)	*P. vivax* n (%)	p-value
Male	571 (56.7)	22 (3.9)	5 (22.7)	17 (77.3)	0.063
Female	436 (43.3)	21 (4.8)	3 (14.3)	18 (85.7)	
Age (yrs)					
<1	192 (19.1)	8 (4.2)	1 (12.5)	7 (87.5)	
1-4	522 (51.8)	22 (4.2)	4 (18.2)	18 (81.8)	0.986
5-14	293 (29.1)	13 (4.4)	3 (23.0)	10 (77.0)	

Malaria was detected in all age groups from 0-14 years. The total number of infants (<1 year) was 192 (19.1%). Among these infants, 8 (4.2%) were found to be slide-positive (1 *P. falciparum*, 7 *P. vivax*). Children aged 1-4 years constituted 51.8% (n=522) of the study population of whom 22(4.2%) were malaria slide-positive (4 *P. falciparum*, 18 *P. vivax*). Of children that belonged to the age group 5-14 years (n=293), 13(4.4%) were slide-positive. The difference in malaria prevalence between the age groups was not statistically significant (p=0.986) although the prevalence was slightly higher for 5-14 years of age (Table 3).

## 4 Discussion

From the retrospective data the highest number of malaria slide-positives was in 2009 and then after a declining trend continued with the lowest number of cases in 2015. In the current cross-sectional survey the overall slide-positivity rate was 4.3% which was lower than the previous year in the same season. This decreasing trend might be due to better awareness within communities about malaria and implementation of control strategies. The results also suggest the effectiveness of control interventions being implemented in the area. Integrated control efforts are underway in the district as part of the nationwide malaria control activities. Nevertheless, the observed prevalence among the children examined in this study is still considerable. Similar studies demonstrated a declining trend of malaria from in different parts of Ethiopia [12,13] although there are reports that showed no substantial decrease in malaria trend in other parts of the country [14].

In the retrospective data the overall number of *P. falciparum* infections was slightly higher than that of *P. vivax* but in the current cross-sectional *P. vivax* was predominant. Most of the cases tended to be mono-infections in both sets of data. In a nearby setting, however, a distinct and opposing seasonal pattern was observed in the relative dominance of the two species [15]. The predominance of *P. vivax* might be due to relapse of dormant liver stages or increased treatment pressure against *P. falciparum*. Whichever the explanation might be the findings demonstrate the increased and probably shifting public health importance of vivax malaria in this locality.

It was observed that children aged 5-14 years had slightly higher malaria prevalence compared to the other age categories although this difference was not significant. This might be due to the fact that children of this age are relatively more exposed to infective mosquito bites compared to younger children that are usually under family protection. Mosquito exposure of under-five children is lower because of maternal care, limited travel, and evening outdoor stays although they are commonly considered as the highest malaria at-risk group. Particularly infants are protected against malaria during the first 6 months of life, largely due to lesser exposure to infective bites besides the role of maternal antibodies and fetal haemoglobin [16]. The study has shown that malaria was more common in male children than their female counterparts, which is harder to explain. Studies suggest that adult females are better protected from parasitic diseases than age-matched males for genetic and hormonal factors (reviewed [17]).

Our findings corroborate the effectiveness of high coverage of national malaria control tools coupled with increased community awareness and subsequent community engagement. A shortcoming of the study is the fact that submicroscopic infections may have been missed and could not be confirmed by PCR. The proportion of febrile children that visited the hospital was highest (70.9%) for the under-fives, indicating non-malarial causes of their fevers. Before the availability of affordable and accurate malaria rapid diagnostic test, in most endemic countries it was presumed that malaria was the cause of fever. Not surprisingly, the proportion of fevers due to malaria was very high in the early 1990s, and the priority was to reduce malaria mortality by any means. But, various reports from different parts of sub-Saharan Africa indicate over-diagnosis and over-treatment of malaria in children in secondary healthcare centres [18,19]. Management of non-malarial fevers is critically important. It is highly desired for malaria surveillance to be based on confirmed rather than suspected cases at all levels of the health system. As malaria control measures expand and the proportion of fevers due to malaria falls rapidly, it becomes increasingly important to track confirmed malaria cases, rather than non-malarious fevers, so that resources can be targeted to areas where problems remain and progress in malaria control is accelerated. Accordingly, the WHO has revised its fever treatment guideline in 2010 to ‘Test and treat’ [20-22].

## 5 Conclusions

Both secondary and primary data showed a declining trend of malaria in Silt’i district and K'ibbet town suggesting possible community awareness about the transmission, symptoms and prevention of malaria and the effectiveness of ongoing control practices. The finding gives a clue that planning for pre-elimination of the disease in the locality may be feasible in the near future. Nevertheless, malaria control interventions must be maintained and scaled-up to sustainably reduce its risk and possibly eliminate it from the locality. One serious limitation of the health record system of the study district was that no age and sex categories were present. Seasonal patterns were also not recorded. These limitations could not allow evaluation of sex, age and seasonal differences in the occurrence of malaria in the district in the years considered. Furthermore, the record system contained only malaria cases and not the total number of patients screened. These major flaws need urgent correction and proper handling of the malaria report system in the district.
